# Uniaxial compression testing and Cauchy stress modeling to design anatomical silicone replicas

**DOI:** 10.1038/s41598-020-68886-3

**Published:** 2020-07-16

**Authors:** Georges Hattab, Tilman Ahlfeld, Anna Klimova, Alexander Koepp, Michael Schuerer, Stefanie Speidel

**Affiliations:** 1 Division of Translational Surgical Oncology, National Center for Tumor Diseases (NCT) Dresden, 01307 Dresden, Germany; 2 German Cancer Research Center (DKFZ), 69120 Heidelberg, Germany; 3 Centre for Translational Bone, Joint and Soft Tissue Research, 01307 Dresden, Germany; 4 Faculty of Medicine, University Hospital Carl Gustav Carus, 01307 Dresden, Germany; 5 Technical University Dresden, 01069 Dresden, Germany; 6 Helmholtz Association/Helmholtz-Zentrum Dresden - Rossendorf (HZDR), 01328 Dresden, Germany

**Keywords:** Soft materials, Medical research, Translational research, Biomedical engineering

## Abstract

Anatomically realistic organ replicas or phantoms allow for accurate studies and reproducible research. To recreate a human kidney, mimicry of the elastic properties of the human kidney is crucial. However, none of the related work addressed the design and development of a kidney phantom using only silicone as material. In contrast to paraffin and hydrogel, silicone is an ideal variant for its extended shelf life, soft-tissue-like feeling, and viscoelastic modularity. To this end, we conducted Uniaxial Compression testing and Cauchy stress modeling. Results indicate that none of the available manufacturer silicone brands are suitable for the task of creating a realistic kidney phantom. Indeed, the tested silicone mixtures in low and high strain fall outside the required approximate target compressive moduli of 20 kPa and 500 kPa, respectively. This work provides a frame of reference for future work by avoiding the pitfalls of the selected ready-made silicones and reusing the reported theoretical and experimental setup to design a realistic replica of the kidney organ.

## Introduction

Anatomically realistic organs replicas provide more consistent results than the use of a living subject or cadaver. Indeed, such a replica of an organ or a phantom not only avoids subjecting a living subject to direct risks but it may also be used for pre-operative planning, surgical training, and structural anatomy learning^1–4^. The benefits of using a realistic phantom aren’t only helpful in validation scenarios (e.g. testing the performance of novel robotic surgical systems) but also extend their utility for the validation of software solutions in biomedical imaging. For instance, in the example of soft tissue deformation, a realistic phantom allows a reasonable evaluation of the software solution before proceeding to clinical trials.

Motivated by these aspects, this manuscript investigates the material testing and modeling phases before designing a silicone based kidney phantom. In the specific scope of a human kidney, related work is rare and attests of the difficulty of accurately measuring its mechanical properties. It is mainly due to the organ’s small size and function, i.e. controlling the fluid and ion levels in the body by excreting any excesses^5^. Yet kidneys are involved in up to 20% of severe abdominal trauma cases and up to 14% of chronic kidney disease^6–8^. This motivates ways to acquire accurate data pertaining to its mechanical properties to help identify mechanisms of injury and/or validate computer vision based systems^9^ for assisted or guided surgery.

In this manuscript, we are concerned with the low and high strain moduli as they reflect the mechanical stiffness of the kidney. That is to say, it defines the relationship between stress and strain in the linear elasticity regime of a uniaxial deformation. However, and in the related work, only two papers have determined this modulus in living patients.

The first work examined four kidneys from human patients who underwent total nephrectomy^10^. Thirty cylindrical renal samples were extracted for Uniaxial Compression (UC) testing. The renal cortex was found with varying values depending on the amount of strain exerted on the tissue. Under low strain, the reported values are 19.6 ± 6 kPa. Whereas in high strain, the values were reported at 530 ± 130 kPa.

The second work adapted the Supersonic Shear Imaging technique to map the in-vivo viscoelastic properties of human renal transplants and compared them to biopsies^11^. In this work, the authors considered the medulla and cortex of the kidney and they defined three experimental groups: control or just after transplantation (9 patients), during the 6 months that follow (9 patients), and 1 year after transplantation (31 patients). Their findings indicated that both the cortex and medulla increase in elasticity when there is fibrosis. Two observers reported median values for both the cortex and the medulla. For the cortex: 21.8 ± 3.0 kPa, 22.0 ± 3.8 kPa. For the medulla: 15.0 ± 1.9 kPa, 15.2 ± 2.8 kPa. Their results complemented each other, where one looked at the cortex and the medulla in vivo, and the other measured the values of the cortex in quasi-static conditions.

Additionally, other related work either measured the elastic moduli on cadaveric kidneys^12^, or developed gelatin gel phantoms of the kidney organ^4,13^. Even though significant progress was made by molding hydrogels like gelatin into a pre-defined shape, several issues remain open such as shelf-life or suitable mechanical properties throughout the entire kidney model. Moreover and in the former case, the reported modulus is not in agreement with the aforementioned work. This is due to their storage at 4$^{\circ }$C, possible necrosis of renal tissue, and blood clotting.

To develop a silicone phantom of the kidney, we propose a two step experimental pipeline as detailed in the methods section: (1). Uniaxial Compression testing and, (2). Cauchy stress modeling. All measured and curated data from the UC testing and the source code for the Cauchy stress modeling and technical validation are openly available^14^.

Fitted normal stress/strain curves of the experimental models for M 4511 ELASTOSIL® mixtures (0, 5, 10) under UC at 5 mm/min, respectively. The curve for silicone mixture 50% is omitted for better visual salience. Indeed, after 55%, silicone mixtures 60 to 75% at increments of 5%, converge to a limit herein represented by a thick red line.

## Results

First, our findings indicate that creating kidney replicas from silicone is a difficult task. Indeed, the main challenge is not only to find a silicone mixture that has high elasticity, but also a mixture that is both non-sticky and non-flaky. We have found that the selected silicones meet these requirements when handled according to the manufacturer’s guidelines for curing and thinning.

**Table 1:** Estimated elastic moduli in low and high strain for 00-10 ECOFLEX®silicone mixtures.

00-10 mixture (%)	E1 (kPa)	E2 (kPa)
0*	80.28	1278.90
5	61.81	470.61
10	67.10	750.14

The 0% mixture refers to the original non-diluted silicone. Dilutions are reported in percentages. Values in low strain (E1) and high strain (E2) are reported in kPa, respectively.

Second, and after performing UC testing, the silicones advertised as corresponding to the target ranges of elastic properties of a human kidney do not fall within the required target compression moduli^10^. Using our knowledge of the bilinear curve of the materials, we estimated the values of low (E1) and high (E2) strain moduli. The values of E1 and E2 are reported for each employed silicone in Tables 1 and 2, respectively. The segmented linear regression analysis enabled us to estimate the change points and fit tangent lines between them. Based on the coefficient of determination ($R^2$), we found that all of the models had an R$^2$ > 98% except for one at 91% (M4511 at 45% mixture). From this, we can conclude that all our models fit very well with the experimental data. The low and high strain moduli are reported for each silicone used in Tables 1 and 2. All estimated values for the change points have a Standard Error, such that $\text {SE} \le 10^{-3}$. The closest silicone mixture we found is the 00-10 mixture at 5% of the ECOFLEX®. That is to say, in low and high strain, the moduli (in kPa) are: $\{\text {E1},\text {E2}\}=\{61.81,470.61\}$.

**Table 2:** Estimated elastic moduli in low and high strain for M 4511 ELASTOSIL®silicone mixtures.

M 4511 mixture (%)	E1 (kPa)	E2 (kPa)
0*	514.69	2238.21
10	373.10	1565.53
20	288.75	1497.01
35	205.27	964.76
45	164.60	1411.10
50	134.52	1673.73
55	139.17	1513.38
60	122.04	1641.85
65	129.88	1481.63
70	108.97	1484.34
75	118.41	1417.60

The 0% mixture refers to the original non-diluted silicone. Dilutions are reported in percentages. Values in low strain (E1) and high strain (E2) are reported in kPa, respectively.

**Figure 1:**
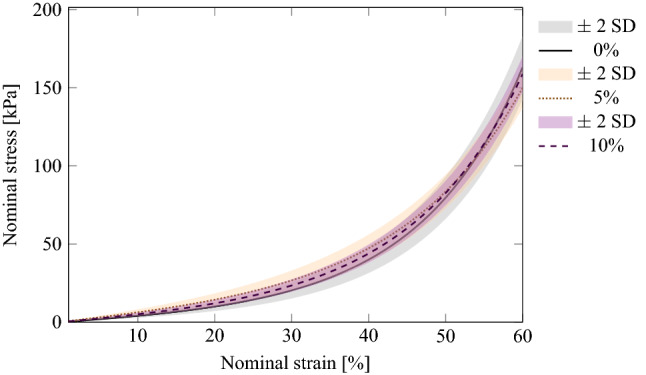
Fitted normal stress/strain curves of the experimental models for 00-10 ECOFLEX® mixtures (0, 5, 10) under UC at 5 mm/min, respectively.

**Figure 2:**
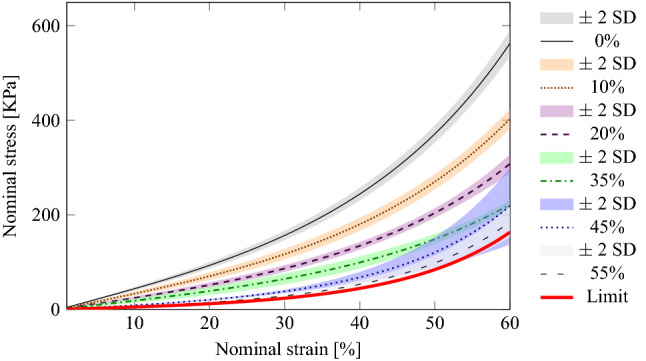
Fitted normal stress/strain curves of the experimental models for M 4511 ELASTOSIL® mixtures (0, 5, 10) under UC at 5 mm/min, respectively. The curve for silicone mixture 50% is omitted for better visual salience. Indeed, after 55%, silicone mixtures 60 to 75% at increments of 5%, converge to a limit herein represented by a thick red line.

Third, Cauchy stress modeling was computed according to the Blatz model. It expresses the constitutive relation between stress and strain in soft tissues. This led to the calculation of two parameters, namely $\alpha$ and $\gamma$. Table 3 lists these parameters for each silicone mixture. Since they characterize each silicone mixture, they are necessary to simulate soft tissue deformation. Figures 1 and 2 show an example visualization of the experimental model (based on the UC testing results) for each silicone manufacturer and each mixture.

**Table 3:** Material parameters for the chosen manufacturer silicones.

Silicone mixture (%)	Alpha ($\mu _\alpha$)	$\text {SD}_{\alpha }$	Gamma ($\mu _\gamma$)	$\text {SD}_{\gamma }$
**00-10 ECOFLEX®**				
0*	2.09	0.09	12.32	2.00
5	1.64	0.12	19.52	3.29
10	1.87	0.09	15.55	1.85
**M 4511 ELASTOSIL®**				
0*	1.05	0.03	144.50	6.08
10	0.99	0.06	110.46	7.46
20	1.03	0.07	80.46	5.24
35	0.99	0.12	61.21	8.36
45	1.64	0.19	27.68	1.63
50	1.96	0.04	16.50	1.42
55	1.81	0.05	19.45	1.63
60	− 14.27	12.25	6.15	6.09
65	1.87	0.05	16.96	0.47
70	− 14.24	12.23	5.57	5.47
75	1.90	0.06	15.40	0.75

The 0% mixture refers to the original non-diluted silicone. Dilutions are reported in percentages. Alpha and Gamma are reported as mean values as the data comprises repeated measures (i.e., technical replicates). For a more comprehensive report, the quantiles of each parameter and silicone mixture are given as TXT files as part of the data repository^14^

Fourth and last, we provide the data and the validation and modeling scripts for reproducibility. An example of a data file obtained after UC test was given as input for Cauchy stress modeling using the Blatz model. The technical validation script is also provided and relies on segmented linear regression analysis and tangential fitting. The data and source code are described in the Materials Cloud Archive^14^.

## Discussion

This work is a stepping stone for future efforts that rely on silicone as a material for creating realistic organ replicas. First, we not only provide the means for reproducibility using openly available data, Cauchy stress modeling and technical validation scripts, but we also provide the community with material parameters to characterize the tested silicone mixtures. The latter could allow the characterization of soft tissue deformations using in silico experiments. This in turn saves time and money before making kidney replicas from silicone.

Second, we argue that in addition to technical validation, errors due to the measurement procedure should also be taken into account. Indeed, the load cell and traverse of the UC test system have both mechanical and electronic errors. Considering the dimensions of the silicone samples tested, these errors could be considered as negligible. Furthermore, this is indirectly modeled in Cauchy-stress modeling, as reported in the Methods and Results sections, respectively.

Third, the thinner concentration affected each silicone differently. For ECOFLEX®, although the data in Table 1 suggests that E2 (high strain) is most affected, we cannot conclude the influence of the thinner due to two factors: (a) the dilution range recommended by the manufacturer is smaller and (b), only three silicone mixtures were considered. For ELASTOSIL®silicone mixtures, the data given in Table 2 suggests that the influence of the thinner stops at 50%; especially with respect to E1 (low strain).

Fourth, in order to estimate E1 and E2, the regression analysis was performed for separate data sets, each accumulating the data for all experiments for the same material type and concentration. The nominal strain range can be divided into four approximate regions where the relationship between stress and strain looks almost linear, but the rate of change in stress appears to be very different. Accordingly, a segmented linear regression model with three change points was fitted to each data set, which allowed us to identify the ranges of low and high strain values in each case and estimate the rate of change over each region. All models demonstrated a very good fit. Indeed, in each model the Standard Errors for the change points did not exceed $10^{-3}$. Figures 1 and 2 report the fitted normal stress/strain curves of the fitted models.

Fifth, the alpha ($\alpha$) and gamma ($\gamma$) parameters reported in Table 3 detail the fit parameters across our experiments. Our work demarks itself from previous work at multiple fronts: (a) our data showcases less variability and less uncertainty, (b) the related work reports an $E_{\mathrm{max}}$ that occurs at a much earlier stage^15^, and (c) we report a larger value for the maximal reachable stress of the tested silicone mixtures.

Sixth, friction plays a role in uniaxial compression test experiments^16,17^. Related work found no significant differences for the chosen silicone^18^. Indeed, finite element (FE) simulation experiments revealed no significant effects in the ranges of a friction coefficient for silicones between 0.6 to 0.9, which corresponds to the range of the tested silicone^18^. Since this neither affects the reaction forces, nor the geometrical changes, our measurements and results are comparable.

Seventh and last, apart from related work dealing with the preparation of organ replicas using paraffin and hydrogel, silicone was so far only been considered in one other related work^12^. Silicone is an ideal variant because of its extended shelf life, its soft-tissue-like feeling and its viscoelastic modularity. Although initially intended, the study of the viscoelasticity isn’t incorporated in this work. However, in none of the related papers was there a clear workflow to successfully reproduce and complete the necessary steps before creating an organ replica. This especially true for the creation of kidney phantoms with silicone as the material. Since these steps are required before forming a negative mold to curate an organ phantom, this manuscript is by large advantageous.

## Methods

Although previous research has shown that the inner (medulla) and outer (cortex) structures of the kidney have slightly different elastic moduli, we focus on reaching the mechanical properties of the cortex. This decision is supported by the fact that the cortex occupies the majority of the organ’s volume. As suggested by the related work, in low strain the renal cortex Young’s modulus is in the interval 13.6 to 25.8 kPa. We define the target required range for the compressive moduli of 20 kPa and 500 kPa in low and high strain, respectively. However, the main challenge is not only finding a silicone mixture that exhibits high elasticity but also one that is both non-sticky and non-flaky. The relationship between stress and strain is a nonlinear function, with elastic modulus being its derivative. For certain ranges of nominal strain this relationship becomes almost linear, and thus the ratio stress/strain is almost constant. It is in fact the slope of the straight line approximating the stress versus the strain function.

To this end, we choose two different silicone types with an identical mixture viscosity of 13 MPa.s: 00-10 ECOFLEX® and RT M 4511 ELASTOSIL®. To find which silicone is closest to life-like elastic properties of a human kidney, different dilutions or mixtures are prepared. This permits us to investigate the range in which a silicone mixture exhibits similar mechanical properties. Hence each silicone type was diluted with a thinner: ECOFLEX®with the Silicone Thinner®/1 by Smooth-On, Inc. to reach 5 and 10% dilutions, ELASTOSIL®with the Wacker Silicone oil AK 100 (and T21 for hardener).

### Sample preparation

To obtain homogeneous mixtures, the silicone is processed according to the manufacturer’s guidelines. This includes handling, dilution, and curation. Mixture preparation was conducted in room temperature, and curation was completed under vacuum. To be comparable to previous work, where the Young’s modulus was measured on cylindrical renal tissue samples, a biopsy punch is used to create cylindrical silicone samples of the exact dimensions (i.e. 7 mm in diameter and 5 mm in height)^10^.

For experimental validation, technical replicates are created. For consistent and cheap production of technical replicates, the silicone is cast into round containers identified by the category ID 9.106 240 (c.f. Gerber and Novelis distributors). The specifications of a container are: capacity of 28 mL, bottom diameter of 51 mm, top diameter of 64 mm, fill height of 13 mm. Indeed, all containers are marked at 5 mm height for casting and the puncher is employed on each container resulting in ten cylindrical samples. Moreover, technical replicates are created for each silicone mixture across manufacturer-specific silicone: $5\times$ ECOFLEX®, $10\times$ ELASTOSIL®.

### Uniaxial compression testing

A Zwick-Roell Z010 equipped with a 100 N load cell (Zwick-Roell, Ulm, Germany) machine was used to conduct the Uniaxial Compression testing (UC) so as to measure the nominal strain/stress. Compression tests are conducted in the axial direction; it relates to the cylindrical direction, where the force is applied on the axis. For the UC, we employ a fixed strain-rate of 5 mm/min for each sample.

### Cauchy stress modeling

For characterization of the material behavior, a nonlinear theoretical simulation based on a two parameter Blatz model is used. Thanks to this model, we can estimate the shape of the stress-strain curves^15^. According to the Blatz model, the constitutive relation, i.e., the relation between the Cauchy stress and the deformation for the soft tissues can be expressed as follows^19^:

1 $$\begin{aligned} \sigma _0 = \dfrac{\gamma }{\alpha +1} \left( \lambda e^{\alpha (\lambda ^2-1)} - \dfrac{1}{\lambda ^2}e^{\alpha \left( \dfrac{1}{\lambda }-1\right) } \right) \end{aligned}$$

In this expression, $\sigma _0$ is the Cauchy stress, and $\lambda$ the ratio of the deformed length to the initial length of the sample. Two material parameters are $\alpha$ and $\gamma$, so the constitutive relation of the material is obtained when these two parameters are determined. The curve fitting parameters in the tangential direction permit the characterization of the kidney tissue by calculating these two material parameters. These parameters characterize the nonlinear elastic model of the chosen silicone mixture. Hence, they are sufficient for the purpose of nonlinear finite element simulation of a whole silicone-based kidney^15^. The parameters are reported in Table 3.

### Technical validation

Technical validation comprises many aspects. First, for the calculations of E1 or the elastic modulus in low strain, we did consider the aspect of linearity of the slope of measurements and its coverage. If it didn’t reach 35% of the compression, the compression value was decreased for all samples to the smallest value. Second, to determine coefficients for the linear slope, measurements were made until 33%. Third, and as used in related work, low and high strain moduli were reported^10^. To find these values, we use our knowledge of the bilinear material curve. In the case of the cylindrical samples tested in UC, there is a nearly linear region (toe) between 0-20% strain. It is followed by a tightly curved region (heel), which is followed by another nearly linear region. The slope of both *linear* regions define the moduli E1 (toe) in low strain and E2 (post-heel) in high strain, respectively. E1 and E2 are the averages obtained from 10 approximations for the segmented line with different random seeds.

The segmented linear regression analysis enables us to estimate the change points and fit tangent lines between them. The estimate for the low strain modulus E1 was, therefore, computed as the slope of the line over the first region (from the 0% strain to the first change point), and the high strain modulus E2 estimate was set equal to the slope of the line over the region between the second and third change points. The regression analysis was carried out using the R package segmented^20^. The initial approximation for the change points was set to the nominal stress of 30%, 40%, and 60%. The estimates for the change points and the corresponding line slopes were obtained as the averages over ten runs with different random seeds of the R function segmented(), where each run was based on n.boot=10 bootstrapping samples. The resulting values for E1 and E2 are reported for each employed silicone in Tables 1 and 2.

## Data records

The data repository is shared as a lossless compressed ZIP file in the Materials Cloud Archive^14^. Each data record associated with this work is included in that file and follows the herein presented hierarchy. The data is shared as primary or raw data and as curated or final data. The data repository includes a LICENSE file, a README.md markdown file, and the data.zip file. 
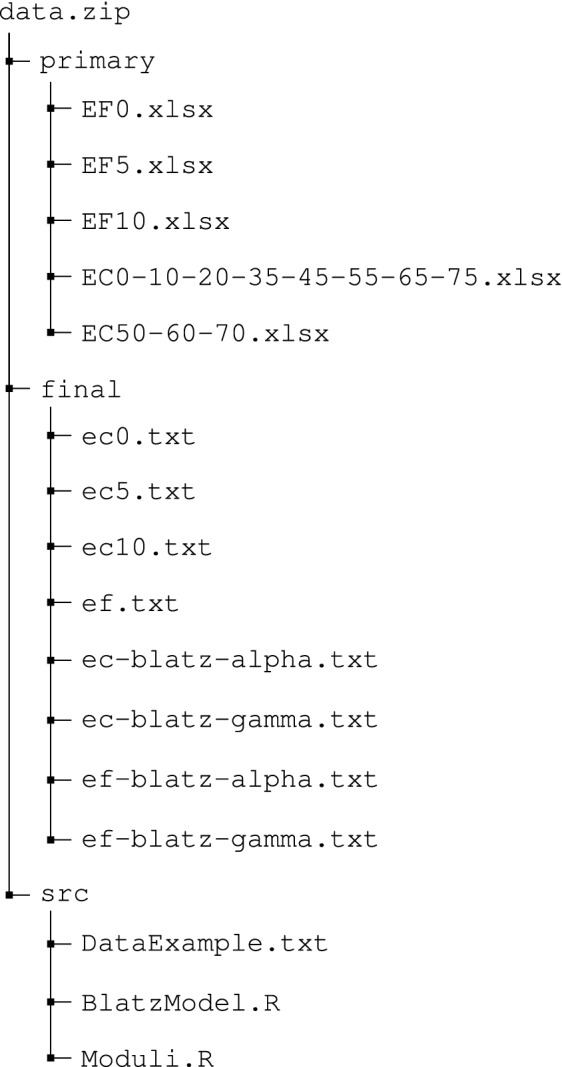


The primary data reports all UC testing and the resulting recorded measurements of stress and strain. Each XLSX or Microsoft Excel Open XML Format Spreadsheet file reports the specified recorded data. Each XLSX file contains two types of spreadsheets: a data spreadsheet for each technical replicate (including a line plot representing the standard force in kPa as function of the deformation in percentage), and a results spreadsheet for a quick summary.

## Usage notes

The provided source code files permit us to format the primary data and to model the Cauchy stress:

- DataExample.txt Example of the primary data obtained after UC. The file contains two columns: nominal strain and stress
- BlatzModel.R computes the Cauchy stress modeling using the Blatz model (c.f. Cauchy stress modeling)
- Moduli.R validates the secondary data using a segmented linear regression analysis and tangential fitting (c.f. Technical validation).

The data and the source code are openly available under Creative Commons Attribution license (CC BY 4.0). Others are free to share, copy, and redistribute the material in any medium or format. They are also free to adapt, remix, transform, and build upon the material. The complete license is provided under the LICENSE file. For More usage related information, please refer to the README file.
